# Risk of esophagus cancer in diabetes mellitus: a population-based case-control study in Taiwan

**DOI:** 10.1186/1471-230X-12-177

**Published:** 2012-12-12

**Authors:** Kao-Chi Cheng, Yu-Lung Chen, Shih-Wei Lai, Pang-Yao Tsai, Fung-Chang Sung

**Affiliations:** 1 Department of Family Medicine, China Medical University Hospital, Taichung, 404, Taiwan; 2 School of Medicine, China Medical University, Taichung, 404, Taiwan; 3 Management Office for Health Data, China Medical University Hospital, Taichung, 404, Taiwan; 4 Department of Public Health, China Medical University, Taichung, 404, Taiwan

**Keywords:** Case-control study, Diabetes, Esophagus cancer, Insulin

## Abstract

**Background:**

Diabetes mellitus (DM) has been associated with the cancer risk. This study investigated relationship between DM and esophageal cancer using Taiwan’s insurance data.

**Methods:**

We identified 549 patients with esophageal cancer newly diagnosed in 2000-2009 and randomly selected 2196 controls without any cancer, frequency matched by sex, age and diagnosis year of cases. Logistic regression model estimated odds ratios (ORs) and 95% confidence intervals (CI) of esophageal cancer associated with DM, sex, age. co-morbidities and medications.

**Results:**

Cases were more prevalent than controls for alcoholism and esophageal disorders and using nonsteroidal anti-inflammatory drugs (NSAIDs) and cyclooxygenase-2 inhibitors but not DM. Esophageal cancer had no association with DM (OR 0.99, 95% CI 0.71-1.37), but significantly associated with alcoholism (OR 14.1, 95% CI 7.87-25.4), esophageal ulcer (OR 10.1, 95% CI 5.70-17.9), esophageal reflux (OR 3.47, 95% CI 2.14-5.26) and uses of NSAIDs (OR 2.73, 95% CI 1.80-4.13). An elevated risk of esophageal cancer appeared in DM patients taking insulin (OR 2.57, 95% CI 1.08-6.15) or sulfonyurea (OR 3.80, 95% CI 1.16-12.5).

**Conclusions:**

Patients with DM are not at higher risk for esophagus cancer. However, esophageal disorders and anti-diabetic drugs are associated with the risk of the disease.

## Background

Glucose metabolism disorders (GMDs) such as diabetes mellitus (DM), impaired fasting glucose regulation (IFG), impaired glucose intolerance (IGT), and hypoglycemia are systemic diseases that have been associated with many other diseases including malignancy [1]. The estimated prevalence of diabetes for all ages worldwide may increase from 2.8% in 2000 to 4.4% in 2030 [2]. DM is also a prevalent public health problem in Taiwan. According to statistics, the mortality rates from DM have almost doubled over the past 10 years in Taiwan. The prevalence of DM in Taiwan has been higher than 5% since 1985 [3]. Studies have associated DM with increased risk of cancers, such as esophageal cancer, hepatocellular carcinoma, bladder cancer, kidney cancer, breast cancer, and endometrial cancer [4-11]. The risk of esophageal cancer linking with DM has been controversy. A meta-analysis, including 6 case-control studies and 11 cohort studies, found a summary relative risk of 2.72 (95% confidence interval (CI) 1.01-4.46) for esophageal cancer linking with DM [8].

Esophagus cancer incidence has been rising rapidly in many countries [12-16]. The incidence of esophageal adenocarcinoma rose approximately sixfold in the United States from 1975 to 2001 [15]. The 5-year mortality rates may exceed 80% for patients with the cancer [17]. Histological data have shown that populations with esophageal cancer in the Western countries suffer mainly from the adenocarcinoma [12-17]. Population in Taiwan has exactly the other type of esophageal cancer; approximately 95% patients have the squamous cell carcinoma [18]. The association between squamous cell carcinoma and DM in the Taiwan population may be different from that between adenocarcinoma and DM in the Western populations. This study was designed to use the Taiwan National Health Insurance claims data to evaluate whether the squamous cell carcinoma of the esophagus is associated with DM.

## Methods

### Data sources

This case-control study used data available from the National Health Insurance (NHI) program in Taiwan. The insurance program was integrated from all public insurance programs in 1995 as a single payment program [19-22]. The National Health Research Institutes are responsible to management the claims data and converted the data into various data sets for research. This study used the claims data of inpatient and out-patients of 1-million insured people randomly selected from all 23-million population. Data files can be linked with scrambled identification to protect individual privacy. Information on anthropometry, laboratory tests and lifestyle was not available.

### Study subjects

This study identified 549 patients aged 20 years and above newly diagnosed with esophageal cancer (ICD-9 150.X and V10.03) during the period of 2000-2009. The index date for each case was the date of diagnosis of esophageal cancer. For each esophageal cancer case, four controls were randomly selected from the same data set frequency matched by sex, age, and then the year the case being selected when controls having a health care visit in the same year. Subjects with esophageal cancer and any other cancer (ICD-9 140-208 and A-code A08x-A14x) identified by the index year were excluded. We retrospectively screened to 1997 for the medical diagnosis of DM (ICD-9 250.xx and A-code A181) with prescription of DM medication for each selected subject. Similar process was performed to screen for co-morbidities that may associate with the development of esophageal cancer. They were alcoholism (ICD-9 codes 303, 305.00, 305.01, 305.02, 305.03, and V11.3 and A-code A215), tobacco use disorders (ICD-9 codes 305.1X), periodontal disease (523.8 and 523.9X), HPV infection (079.4), hyperlipidemia (272.0, 272.1, 272.2, 272.3 and 272.4), Barrett's esophagus (530.85), esophageal ulcer (530.2), esophageal reflux (530.81, 530.11) and esophageal burn (947.2). Other factors examined were the use of nonsteroidal anti-inflammatory drugs (NSAIDs) like aspirin, cyclooxygenase-2 inhibitors (COX-2 inhibitors) and anti-diabetic drugs identified from the prescription files. The anti-diabetic drugs included metformin, sulfonylureas, thiazolidinediones, alpha-glucosidase inhibitors, D-phenylalanine derivatives, dipeptidyl peptidase 4 inhibitors, incretin mimetic agents and insulins.

### Statistical analysis

We compared the distributions in sex, age, co-morbidities and medications between esophageal cancer cases and controls and tested differences using the Chi-square test. The significant variables were further included in the multivariate logistic regression analysis to measure odds ratio (OR) and 95% CI for esophageal cancer, with diabetes status forced in the analysis. Further analyses explored whether the risk of esophageal cancer was associated with the duration of diabetes (< 2 vs. ≥ 2 years) and using anti-diabetic drugs. The association with anti-diabetic drug was estimated individually. The statistical significance level was set at probability value of < 0.05 (SAS software version 9.1, SAS Institute Inc., Cary, North Carolina, USA).

### Ethical considerations

This study used surrogate identification for each study subject to link study files to secure patient privacy. The present study was exempted from a full ethical review.

## Results

### Baseline characteristics

Table 1 compares the demographic characteristics and co-morbidities between esophageal cancer cases and controls. Both groups were predominantly men (92.9%) with the mean age of 60.9 (standard deviations 3.03 vs. 12.9) years. There were no significant differences between cases and controls in the prevalence of DM (10.4% vs. 10.1%, p = 0.85) and hyperlipidemia. Cases were more prevalent with co-morbidities of alcoholism, esophageal ulcer, esophageal reflux, and COX-2 inhibitors except use of other NSAIDs.

**Table 1:** Comparisons in socio-demographic status, co-morbidity and medication between esophageal cancer cases and controls frequency matched by sex, age and index year

	**Esophageal cancer**				
	**No**		**Yes**		
	**N=2196**		**N=549**		
	**n**	**%**	**n**	**%**	***P *value**^**†**^
Age, mean (SD), years	60.9	(3.03)	60.9	(12.9)	0.24
20-39	56	2.55	14	2.55	1.00
40-64	1,328	60.5	332	60.5	
≥65	812	37.0	203	37.0	
Sex					1.00
Women	156	7.10	39	7.10	
Men	2,040	92.9	510	92.9	
Comorbidity					
Diabetes mellitus	222	10.1	57	10.4	0.85
Alcoholism	16	0.73	53	9.65	<0.0001
Obesity	4	0.18	1	0.18	1.00^†^
Tobacco use disorder	7	0.32	5	0.91	0.07^†^
Periodontal disease	33	1.50	2	0.36	0.03^†^
HPV infection	--	--	--	--	--
Hyperlipidemia	324	14.8	65	11.8	0.08
Barrett's esophagus	--	--	--	--	--
Esophageal ulcer	18	0.82	50	9.11	<0.0001
Esophageal reflux	39	1.78	43	7.83	<0.0001
Medication					
Use of aspirin	305	13.9	83	15.1	0.46
Use of COX-2 inhibitors	495	22.5	159	29.0	0.002
Use of other NSAIDs	1,877	85.5	522	95.1	<0.0001

Data are presented as the number of subjects in each group, with percentages given in parentheses.

Chi-square test and ^†^Fisher’s exact test comparing patients with and without esophageal cancer.

### Risk measures

Table 2 shows that esophageal cancer was not related to DM in both univariate and multivariate logistic regression analyses (OR 0.99, 95% CI 0.71-1.37). Among co-morbidities, esophageal cancer had the strongest association with alcoholism (OR 14.1, 95% CI 7.87-25.4), followed by esophagus ulcer (OR 10.1, 95% 5.70-17.9), esophagus reflux (OR 3.47, 95% CI 2.14-5.62) and use of other NSAIDs (OR 2.73, 95% CI 1.80-4.13). Table 2 also shows a protective association between periodontal disease and esophageal cancer (OR 0.20, 95% CI 0.02-0.88).

**Table 2:** Crude and adjusted odds ratios and 95% confidence intervals of esophageal cancer associated with diabetes and covariates

	**Crude**		**Adjusted**	
**Variable**	**OR**	**(95%CI)**	**OR**	**(95%CI)**
Diabetes mellitus				
No	1.00		1.00	
Yes	1.03	(0.76-1.40)	0.99	(0.71-1.37)
Alcoholism				
No	1.00		1.00	
Yes	14.6	(8.25-25.7)	14.1	(7.87-25.4)
Periodontal disease				
No	1.00		1.00	
Yes	0.24	(0.06-1.00)	0.20	(0.04-0.88)
Esophageal ulcer				
No	1.00		1.00	
Yes	12.1	(7.01-21.0)	10.1	(5.70-17.9)
Esophageal reflux				
No	1.00		1.00	
Yes	4.70	(3.02-7.33)	3.47	(2.14-5.62)
COX-2 inhibitors				
Never use	1.00		1.00	
Ever use	1.40	(1.14-1.73)	1.06	(0.83-1.34)
Other NSAIDs				
Never use	1.00		1.00	
Ever use	3.28	(2.19-4.92)	2.73	(1.80-4.13)

Adjusted for sex, age, DM, alcoholism, periodontal disease, esophagus ulcer, esophagus reflux, other NSAIDs and COX-2 inhibitors.

Table 3 shows patients with DM for longer than 2 years were at higher risk of the cancer than patients with shorter DM history with an OR of 2.28 (95% CI 0.85-6.08) after controlling for sex, age, alcoholism, periodontal disease, esophagus ulcer, esophagus reflux, COX-2 inhibitors and other NSAIDs.

**Table 3:** Adjusted odds ratios for esophageal cancer in relation to duration of diabetes mellitus

**Duration of diabetes (years)**	**Case**	**Control**	**Odds ratio (95% CI)**	
	**Number (%)**		**Model 1**	**Model 2**
0-2	6	44	1.00	1.00
≥2	51	178	2.10 (0.85-5.31)	2.28 (0.85-6.08)

Model 1 was adjusted for age and sex.

Model 2 was additionally adjusted for sex, age, alcoholism, periodontal disease, esophagus ulcer, esophagus reflux, other NSAIDs and COX-2 inhibitors.

### Influence of anti-diabetic drugs

Data analysis showed that patients taking anti-diabetic drugs were at higher risk of esophageal cancer (Table 4). The associations were significant for insulin (OR 2.58, 95%1.08-6.15) and sulfonylureas (OR 3.80, 95% 1.16-12.5), and moderately significant for metformin.

**Table 4:** Odds ratios of esophageal cancer in relation to use of anti-diabetic drugs

**Use of anti-diabetic drugs**	**Case**	**Control**	**Odds ratio (95% CI)**	
	**Number (%)**		**Model 1**	**Model 2**
Insulins				
never use	7(12.3)	69(31.1)	1.00	1.00
ever use	50(87.7)	153(68.9)	3.27(1.40-7.60)	2.58(1.08-6.15)
Metformin				
never use	5(8.77)	43(19.4)	1.00	1.00
ever use	52(91.2)	179(80.6)	2.34(0.87-6.24)	2.84(0.99-8.18)
Sulfonylureas				
never use	4(7.02)	38(17.1)	1.00	1.00
ever use	53(92.9)	184(82.9)	2.75(0.94-8.10)	3.80(1.16-12.5)
Thiazolidinediones				
never use	49(86.0)	180(81.1)	1.00	1.00
ever use	8(14.0)	42(18.9)	0.65(0.28-1.49)	1.11(0.46-2.65)
Alpha-glucosidase inhibitors				
never use	48(84.2)	188(84.7)	1.00	1.00
ever use	9(15.8)	34(15.3)	1.00(0.44-2.24)	

Model 1 was adjusted for age and sex.

Model 2 was additionally adjusted for sex, age, alcoholism, periodontal disease, esophagus ulcer, esophagus reflux, other NSAIDs and COX-2 inhibitors.

## Discussion

Studies investigating the relationship between malignancy and DM have revealed conflicting results on the association between DM and esophagus cancer, and other cancers [8,23-39]. Based on histology, the distribution of squamous cell carcinoma and adenocarcinoma varies among different geographic areas. Most of studies on esophageal cancer have been conducted on adenocarcinoma of the esophagus for the populations in Western countries [8,23-42]. The positive association between the risk of esophagesl cancer and DM in the meta-analysis conducted by Huang et al consisted of 15 studies from Western countries and two studies from Asian countries [8]. More than 80% of esophagus cancer cases in the Asian are squamous cell carcinoma [18]. Whether the risk of squamous cell carcinoma of esophagus has a relationship with DM has not been well explored. Our study makes up for the gap because the previous study found that the squamous cell carcinoma is the major type of esophageal cancer in Taiwan, accounting as high as 95% of patients with the disease [18].

The present population-based case-control study failed to find a significant association between DM and esophagus cancer in Taiwan. Regardless no association was found between DM and esophageal cancer, this study shows several co-morbidities were much more prevalent in cases than in controls. Consistent with other studies [26,27,37-39], we also found heavy alcohol drinking is the strongest risk factor associated with esophageal cancer. A Korean study found a relative risk of 5.62 for mortality from esophageal cancer [26]. Japanese have an OR of 15 for esophageal caner among high alcohol consumers [27]. The risk may increase further to approximately 70-fold higher for those with alcohol flushing response, indicating individuals with difficulty to metabolite alcohol are at a much higher risk for the cancer. The exact mechanism of how alcoholic beverage is associated with esophagus cancer risk is not clear. Although ethanol has not been shown as carcinogenic in laboratory animals, it may act through its major metabolite, acetaldehyde, a carcinogen in animal models [27,38,43-45]. The dose-response relationship with alcohol strongly indicates it as an independent risk factor, particularly for esophagus squamous–cell cancer [26,37,39].

Esophageal cancer is significantly associated with esophageal ulcer and esophageal reflux, and medications in this study. Both esophageal ulcer and esophageal reflux are likely the earlier signals for the subsequent development of the cancer of esophagus due to esophageal stricture formation, such as acid peptic and medication-induced. Peptic strictures account for most of esophageal strictures [46-49]. Purdy et al. found esophageal mucosa injury is associated with sloughing esophagitis, chronic debilitation and medications [46]. García Rodríguez et al. have also reported that long term pharmacological gastric acid suppression is associated with the risk of oesophageal and gastric adenocarcinoma [47]. In this study, anti-diabetes drugs and other NSAIDs are significantly associated with the disease.

### Limitations

This study was conducted using insurance claims data, a few limitations should be considered. First, smoking is an important factor associated with esophageal cancer. But, this information is not available in the insurance claims. However, we are able to identify patients with the diagnosis of alcoholism. There is strong relationship between drinking and smoking. And we did find alcoholism is an indicator that may well predict the cancer. Second, this case-control study enrolled study subjects with and without esophageal cancer identified from 2000 to 2009. The frequency matched study design fails to observe age difference between cases and controls. However, we have also conducted an unmatched analysis by randomly sampling controls, which revealed a mean age of 15.4 years younger in the controls. This analysis also showed that DM is not a factor associated with esophageal cancer. Therefore, the frequency matched samples are representative of the insured population. Finally, the insurance registry does not provide the information on histological type and genotype of the cancer. We were unable to differentiate whether the associated risk factors different between squamous cell carcinoma and adenocarcinoma among study subjects. It is likely that the small portion of adenocarcinoma may not change the association between DM and the risk of esophageal cancer in the present study.

## Conclusions

In conclusion, the results of our study fail to show a significant association between DM and the risk of esophagus cancer. Instead, alcoholism, esophageal ulcer and esophageal reflux are significant predictors for the cancer. Patients of heavy drink, with esophageal ulcer, esophageal reflux and peptic strictures are at high risk of developing the disease.

## Competing interests

The authors disclose no conflicts of interest.

## Authors’ contribution

KCC: (1) substantial contributions to the conception of this article; (2) planned and conducted the study; (3) initiated the draft of the article and critically revised it; S-WL: (1) substantial contributions to the conception of this article; (2) planned and conducted the study; (3) initiated the draft of the article and critically revised it; Y-LC: (1) substantial contributions to the conception of this article; (2) initiated the draft of the article and critically revised it; P-YT: (1) conducted data analyses; (2) critically revised the article; F-CS: (1) substantial contributions to the study concept and design; (2) conducted data analysis and data interpretation; (3) critically revised the article. All authors read and approved the final manuscript.

## Pre-publication history

The pre-publication history for this paper can be accessed here:

http://www.biomedcentral.com/1471-230X/12/177/prepub
